# No single place for space: neuronal representation of location beyond the hippocampus: Comment on: Sauer JF, Folschweiller S, Bartos M (2022) Topographically organized representation of space and context in the medial prefrontal cortex. PNAS 119:e2117300119

**DOI:** 10.1007/s00424-022-02699-3

**Published:** 2022-05-07

**Authors:** Andreas Draguhn

**Affiliations:** Institute for Physiology and Pathophysiology, Medical Faculty, Heidelberg University, Im Neuenheimer Feld 326, 69120 Heidelberg, Germany

The perception, processing, and recall of space- and context-related information has become a major topic in mammalian cognitive neurosciences, following several milestone discoveries: the location-specific activation of “place cells” [1], the entrainment of such cells into temporal sequences by network oscillations [2], the re-play of such sequences during slow-wave sleep [3], and complex spatial tuning properties of “grid cells,” “head direction cells,” or “border cells” [4]. Together, we have gained considerable insight into the neuronal underpinnings of spatial cognition.

There are, however, major gaps in our knowledge. First, the mechanisms underlying long-term consolidation of spatial memories are largely unknown. Second, the physical substrate of remote memories and the mechanisms of their re-activation are elusive. Third, the functions and interactions of different brain areas in spatial cognition are highly under-studied. In order to answer these questions several groups go beyond the traditional focus on the hippocampal formation and include further areas which are involved in spatial information processing: medial and lateral entorhinal cortex, peri- and postrhinal cortex, frontal, prefrontal, parietal and somatosensory neocortex, ventral and dorsal striatum, thalamic nuclei, and other subcortical areas. This development is not at least fostered by technological progress in structural and functional connectomics and in long-term high-resolution recordings from awake, behaving animals.

Sauer, Folschweiller, and Bartos [5] have now added an important piece of information to the current scenario of distributed spatial information processing. They focused on the medial prefrontal cortex (mPFC) of mice, a highly interconnected association cortex required for cognitive and behavioral flexibility, decision making, and control of executive functions [6]. The mPFC expresses marked functional specializations with emotional processes preferentially represented in ventral and cognitive content in dorsal portions. A similar dorsal-to-ventral gradient is present in the hippocampus: ventral portions are strongly involved in emotional processing, have larger place fields, and may contribute less to spatial memory formation [7, 8]. Interestingly, hippocampal projections to the mPFC are concentrated in ventral parts of both areas [9], indicating a preferential role in emotional and motivational processes. On the other hand, space-specific tuning of neurons has been previously shown in all parts of the mPFC, including dorsal portions [10].

**Fig. 1.**
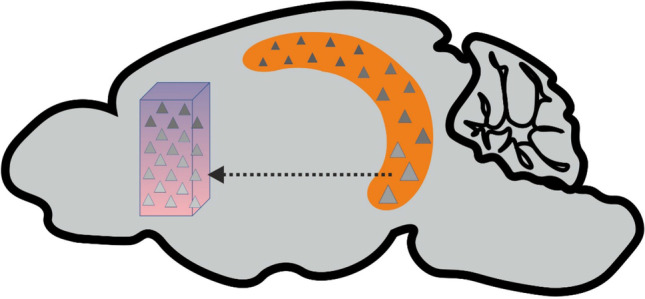
Different topologies of spatial information processing in the mouse medial prefrontal cortex (pink) and hippocampus (orange). Triangles represent individual neurons with different depth and stability of spatial tuning (dark grey indicating strong tuning). Different sizes of triangles in the hippocampus represent differently sized place fields. Schematic brain envelope taken from Emmett Thompson (Zenodo; 10.5281/zenodo.3925987, 2020)

In this situation, Sauer and colleagues analyzed spatial tuning properties of neurons throughout the dorsal-ventral axis of the mPFC. Using implantable electrodes with multiple (64) linearly arranged contact sites they were able to record action potentials from differently located, identified neurons over prolonged periods (technically, these potentials are called “unit discharges” and the sites of origin “units” because the individual neurons cannot be morphologically reconstructed with this extracellular recording technique). The behavioral setup consisted of a circular track, presented as virtual reality, while the animal was head-fixed and running on a wheel. This setting restricts the animal’s behavior and allows fast switches between different “environments,” facilitating interpretation of data. There was also no reward or emotional load by aversive stimuli, such that the experiment merely addressed spatial information processing during the natural exploration behavior of the animals. With this approach, they made three important discoveries: (i) about one third of all neurons in the mPFC are active at specific locations (i.e., show spatial tuning); (ii) changing the virtual environment rapidly changes neuronal tuning, a process called remapping which had, hitherto, only be observed in the hippocampus; and (iii) spatial tuning is stronger in dorsal than in ventral regions of the mPFC. This finding suggests that the dorsal mPFC is particularly important to convey spatial information to downstream motor networks, supporting well-adapted movements in the environment. At the same time, the functional gradient is exactly opposite to the reported distribution of neuronal connections from the hippocampus, raising the question of its origin. In any case, the unexpected topology of neuronal tuning suggests that spatial information arrives in the medial prefrontal cortex more independently from the hippocampus than previously thought (Fig. 1). This notion could indicate that positional cues are processed in a highly parallel and distributed way, rather than in a hierarchical order of concatenated networks. The matrix of networks could then support differential behavioral or cognitive reactions to different dimensions of spatial information: the own localization, an allocentric map of the environment, planning of foraging, expected reward or punishment, place-associated sensory (e.g., olfactory, tactile) information, non-spatial elements of context, etc. [11]. This systemic perspective goes beyond any naive locationalism and may explain why disruption of signals from “higher” associative networks like the mPFC impair spatial tuning of neuronal activity in more basic or “lower” areas like the hippocampus [10].

Finally, the findings by Sauer, Folschweiller, and Bartos remind us that we rely on precise structural and functional data from multiple brain regions. Combined with the increasingly powerful interventional methods this may enable us to generate concrete, causal models of information processing and behavioral control. This endeavor also requires a cross-species, comparative approach, especially with respect to the massive changes in size and structure of prefrontal areas during mammalian evolution. There is a lot of work ahead, and the tools are all there!
